# The Associations between Caregivers’ Emotional and Instrumental Feeding, Children’s Emotional Eating, and Children’s Consumption of Ultra-Processed Foods in China

**DOI:** 10.3390/ijerph19084439

**Published:** 2022-04-07

**Authors:** Meijing An, Xiyao Liu, Hao Guo, Qianling Zhou

**Affiliations:** 1 Department of Maternal and Child Health, School of Public Health, Peking University, Beijing 100191, China; mj_an1128@bjmu.edu.cn (M.A.); liuxiyao1997@163.com (X.L.); 2 Loudi Town Health Centre, Luancheng District, Shijiazhuang 051430, China; lcqgh880317@163.com

**Keywords:** emotional feeding, instrumental feeding, emotional eating, ultra-processed foods, infants and toddlers

## Abstract

High consumption of ultra-processed foods (UPF) increases the risks of non-communicable diseases and all-cause mortality in adulthood, and the risk of early childhood caries. Limited evidence about factors influencing children’s consumption of UPF exists. This study was conducted to assess the prevalence of UPF consumption among children less than three years of age, and identify its associations with caregivers’ emotional and instrumental feeding, and children’s emotional eating. A cross-sectional study was conducted in Shijiazhuang, Hebei Province, China. Caregivers caring for children aged 6–36 months (*n* = 408) were recruited. Caregivers’ emotional and instrumental feeding practices and children’s emotional eating were assessed by the Parents’ Feeding Practices Scale for Infant and Young Child and Children’s Eating Behaviour Questionnaire, respectively. Children’s UPF consumption was assessed by a validated Food Frequency Questionnaire. Of the children, 86.8% consumed UPF. The highest percentage of UPF consumed was pastries (63.5%), followed by solid or semi-solid dairy products (58.8%), and reconstituted meat products (56.4%). Caregivers’ emotional and instrumental feeding was positively associated with children’s consumption of UPF (OR = 1.59, 95%CI: 1.01, 2.49), a higher frequency of UPF consumption weekly (OR = 1.80, 95%CI: 1.35, 2.39), and a larger amount of UPF consumption weekly (OR = 1.85, 95% CI: 1.38, 2.49). Children’s higher frequency of emotional undereating was associated with their UPF consumption (OR = 1.61, 95%CI: 1.07, 2.42) and a higher frequency of UPF consumption weekly (OR = 1.33, 95%CI: 1.03, 1.73). Children’s emotional undereating significantly mediated the associations between caregivers’ emotional and instrumental feeding and children’s consumption of reconstituted meat products. Caregivers should be educated to avoid emotional and instrumental feeding practices, and cultivate children’s good eating habits to improve children’s diet quality.

## 1. Introduction

Ultra-processed foods (UPF), defined within the NOVA food classification system [1], are foods made through a series of industrial processes with complex equipment and technologies to process substances, seasonings, and additives into delicately packaged foods [2]. Sugar-sweetened beverages, packaged snacks, and candies are examples of UPF [2]. UPF is palatable, convenient, profitable, and has a long shelf life [2]. UPF is high in energy density, carbohydrates, saturated fatty acids, trans fatty acids, and sodium, and low in fibre, protein, and micronutrients [3,4]. Higher UPF consumption was associated with the risks of metabolic syndrome and mortality among adults [5], higher blood lipid levels among children/adolescents [6], and dental caries among younger children [7]. Eating habits formed in early childhood can persist into adulthood [8,9]. A longitudinal study demonstrated the percentage of energy intake from UPF among children at 24 months was related to that among children at 60 months [10]. A reduction of UPF consumption in early childhood is beneficial for one’s health in the whole life course.

The percentage of UPF consumption was high among young children. In Brazil, 74.3% of children under two years old consumed UPF [11]. In Indonesia, 81.6% of children under three years old consumed packaged snacks, and 40.0% consumed sugar-sweetened beverages [12]. In the US, 85% of children aged two-to-three years old consumed sugar-sweetened beverages, desserts, sweets, or salty snacks daily [13]. In China, according to United Nations International Children’s Emergency Fund, the percentage of sales of dried baby foods and prepared baby foods per capita among children under five years old increased from 2014 to 2019 [14]. However, research on UPF consumption among children under three years of age is still lacking in China.

Caregivers’ feeding practices influence children’s dietary intake. Emotional feeding indicates using foods to calm or soothe, or for boredom or fussiness, and instrumental feeding indicates using foods to reward or punish [15,16]. Caregivers usually provide energy-dense, palatable, and nutrient-poor foods, such as candies and cookies, when they adopt emotional and instrumental feeding [16]. Studies in the Netherlands demonstrated parents’ emotional and instrumental feeding was positively related to children’s energy-dense snack consumption at six-to-nine years old [17,18,19]. In the UK, children whose mothers frequently used emotional feeding strategies ate more cookies and chocolates from three to five years old than children whose mothers did not tend to use emotional feeding [20]. Fathers’ emotional and instrumental feeding was positively associated with sugar-sweetened beverage consumption among Hispanic children aged two-to-five years old in the US [21]. In addition to snacks and sugar-sweetened beverages, the associations between caregivers’ emotional and instrumental feeding practices and children’s consumption of other UPF food items (e.g., reconstituted meat products) need to be further explored.

Children’s eating behaviours are also related to their dietary intake. Emotional eating refers to eating in response to negative emotions such as worry, anger, stress, and depression [22]. Emotions influence differently on appetite, with some experiencing an increased desire to eat (i.e., emotional overeating, EOE), and some experiencing a decreased desire to eat (i.e., emotional undereating, EUE) [23,24]. A study conducted in 12 countries demonstrated emotional eating among children aged nine-to-eleven years old was positively associated with an unhealthy dietary pattern characterised by high loadings for fast foods, ice cream, fried food, cakes, and sugar-sweetened sodas [25]. There is a lack of studies investigating the association between emotional eating and UPF consumption among children under three years old, a critical period of the development of one’s dietary habits. Caregivers’ feeding practices might influence children’s eating behaviours [26], and further play an indirect role in children’s dietary intake [21]. However, evidence examining the mediation effect of children’s eating behaviours on the association between caregivers’ emotional and instrumental feeding and children’s UPF consumption is still lacking.

The aim of this study was to assess the prevalence of UPF consumption among children under three years old in China, and to identify its association with caregivers’ emotional and instrumental feeding, and children’s emotional eating. The mediation effect of children’s emotional eating was also examined.

## 2. Materials and Methods

### 2.1. Study Design

This study was a cross-sectional survey to examine caregivers’ feeding practices and their children’s dietary intake and behaviour in China.

### 2.2. Participants

The caregiver was the primary person responsible for child-feeding daily. The inclusion criteria were: caregivers had children aged 6-to-36 months old, of normal birth weight (i.e., 2500 g ≤ birth weight < 4000 g), of term delivery (i.e., 37 weeks ≤ gestational age < 42 weeks), living in Shijiazhuang, Hebei Province; and caregivers had good literacy and comprehension. The exclusion criteria were: caregivers had serious diseases (e.g., cancer, liver disease, kidney disease, AIDS, or depression), or had children with congenital diseases, infectious diseases, inherited metabolic diseases, dysmorphia, fever, or diarrhea. This study complied with the guidelines in the Declaration of Helsinki, and was approved by the Institutional Review Boards of the Peking University (IRB00001052-20047). Written informed consent was obtained from all caregivers.

### 2.3. Data Collection

Data were collected in a town health centre and village clinics located in Shijiazhuang city, Hebei Province, China, between August and October 2020. A convenience sampling approach was used to recruit the participants. In the town health centre, caregivers who took their children for health check-ups or vaccination were approached and assessed for eligibility. In the village clinic, the village doctor contacted the targeted caregivers by telephone calls, and required them to come to the clinic together with their children if they were willing to participate in the survey. The eligibility of caregivers was assessed according to the inclusion criteria by trained research staff. The eligible caregivers were required to complete a survey through face-to-face interviews by trained research staff. The interview lasted approximately 20 min. After finishing the survey, the completeness of the data was checked, and a toy (approximately 5 CNY) was given to the participant.

### 2.4. Instruments

The questionnaire was used and included questions about the sociodemographic information of caregivers and their children, caregivers’ feeding practices, children’s eating behaviours, and children’s food frequency questionnaire (FFQ).

#### 2.4.1. Sociodemographic Information

Sociodemographic information included caregiver’s education level, employment status, monthly household income, self-reported height and weight, caregiver’s relationship with the child, child’s age, and gender. Caregiver body mass index (BMI) was calculated. According to the criteria of adults’ weight status in China, caregivers’ BMI were classified as underweight, normal weight, overweight, and obese, respectively [27].

#### 2.4.2. Caregivers’ Emotional and Instrumental Feeding Practices

Caregivers’ emotional and instrumental feeding was assessed by the subscale of Parents’ Feeding Practices Scale for Infant and Young Child (PFPSIYC). The PFPSIYC was developed to measure the feeding behaviours of Chinese caregivers with children aged 6-to-24 months, with acceptable validity and reliability (content validity index: 0.910, construct validity: χ^2^/df = 2.00, comparative fit index (CFI) = 0.915, non-normed fit index (NNFI) = 0.904, root mean square error of approximation (RMSEA) = 0.046; Cronbach’s α = 0.783) [28]. The subscale contained five items (Table S1). Participants responded on a 5-point Likert scale (1 = never, 2 = rarely, 3 = sometimes, 4 = often, 5 = always). The average score of the subscale was calculated, and a higher score indicated a higher frequency of using the feeding practices. All items in the PFPSIYC were included into exploratory factor analysis conducted in the current sample. There were seven factors with eigenvalues more than one, which explained 55.5% of variances. The first factor explained 10.6% of variance, and contained five items with loading more than 0.4. These five items were same as the items in the emotional and instrumental feeding subscale of the original PFPSIYC. The subscale showed good internal reliability among the current sample (Cronbach’s α: 0.768).

#### 2.4.3. Children’s Emotional Eating Behaviours

Children’s emotional eating behaviours were measured by the EOE subscale and EUE subscale of the Children’s Eating Behaviour Questionnaire (CEBQ) (Table S1). The CEBQ is a validated and world-widely-used tool reported by caregivers to measure their children’s eating behaviour using a 5-point Likert scale (1 = never, 2 = rarely, 3 = sometimes, 4 = often, 5 = always) [29]. The average scores of the EOE and EUE subscales were calculated, respectively. A higher score indicated a higher frequency of engaging in the eating behaviour. The Chinese translated version the CEBQ had good internal and test–retest reliability among caregivers of primary school children [30], and had good internal reliability (Cronbach’s α: 0.52–0.80) among caregivers with children aged 12–18 months old [31]. In the current study, the Cronbach’s α of the EOE and EUE subscales was 0.698 and 0.733, respectively.

#### 2.4.4. Children’s UPF Consumption

Children’s food consumption in the last week was measured by the FFQ (29 items), which had been used in the Chinese Nutrition and Health Surveillance of children aged 0–2 years old in 2013 [32]. Caregivers reported whether their children consumed each food item, and times of consumption over the last week, and the average amount of food consumed each time. A food atlas including portion sizes of familiar foods eaten by local children was provided to facilitate the estimation of the amount of food consumed. The formula of times of food consumption in the last week multiplied by the average amount of each consumption was used to calculate the amount of food consumed in the last week by children.

All food items in the FFQ were categorised as UPF and non-UPF based on the NOVA food classification system [2] by two researchers (M.A. and X.L.) independently. The consistency of categorization was 90% (26/29). Any inconsistency was discussed with a third researcher (Q.Z.) to reach an agreement. In the present study, the UPF contained (1) sugar-sweetened beverages (e.g., carbonated drinks, fruit juice beverages, tea drinks, milk beverages, flavoured water drinks), (2) solid or semi-solid dairy products (e.g., cheeses, thick yogurts), (3) pastries (e.g., cakes, cookies, caramel treats, chocolate pies with egg yolk, waffles), (4) savoury packaged snacks (e.g., rice cakes, bugles), (5) confectioneries (e.g., candies, chocolates, jelly, hawthorn strips, preserved fruits), and (6) reconstituted meat products (e.g., hams, sausages). The consumption of UPF was defined as the consumption of at least one of the above UPF food items. The times of UPF consumption were defined as the summary of times of the above UPF food items that were consumed. The amount of UPF consumption in the last week was defined as the summary of the amount of the above UPF food items that were consumed. For the sugar-sweetened beverages, the amount in mL was transformed into g according to the estimated density of 1 g/mL.

### 2.5. Sample Size Estimation

Based on a correlation coefficient of 0.21 [33] in the relationship between emotional feeding and the frequency of pastry consumption among children aged one-to-three years old, and considering a power of 80% and a type 1 error of 5%, the sample size requirement for this study was 175. With a consideration of a 10% nonresponse rate, the sample size in this study was estimated to be 184.

### 2.6. Statistical Analysis

In the descriptive analysis, means and standard deviations (SD) were used to describe the normally distributed variables, and frequencies and percentages were used to illustrate the categorical variables. The frequencies and amount of UPF consumption per week were displayed as the median and interquartile range, respectively. The median values were further used to categorise the times and amount of UPF consumption into two groups. The associations between children’s UPF consumption and participants’ sociodemographic characteristics were assessed by chi-squared test. Variables with a *p* < 0.2 were adjusted as potential confounders. The independent effects of caregivers’ emotional and instrumental feeding and children’s emotional eating on children’s UPF consumption were calculated by multivariable logistic regression analyses. The relationships between caregivers’ emotional and instrumental feeding and children’s emotional eating were assessed by Pearson correlation analysis. The mediation effect of children’s emotional eating on the relationships between caregivers’ emotional and instrumental feeding and children’s UPF consumption was explored by the mediation package in R. All statistical analyses were performed by SPSS version 22.0 (IBM Corp., Armonk, NY, USA) and R version 3.6.2. Statistical significance was set at a *p* < 0.05.

## 3. Results

### 3.1. General Characteristics of the Participants

**Figure 1:**
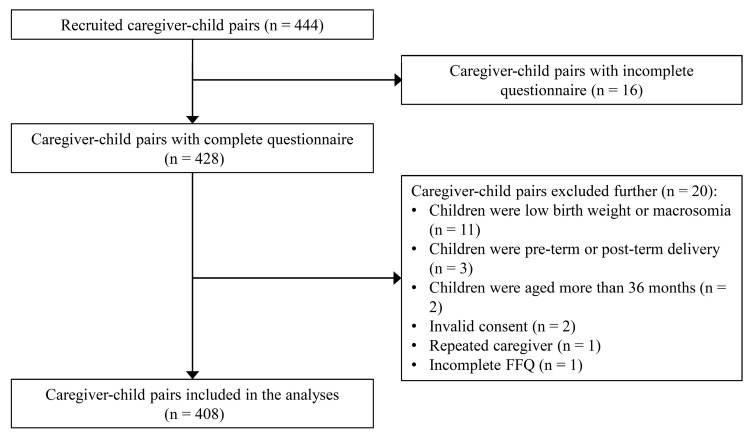
Flowchart of the participants.

A total of 444 caregivers agreed to participate in this study, and 428 of them completed the questionnaire. Twenty caregiver–child pairs were excluded further. In total, data from 408 caregiver–child pairs were analysed (Figure 1). All caregivers included 309 parents (97.1% of those were mothers) and 99 nonparents (94.9% of those were grandmothers). The mean age was 30.2 (SD 3.9) years old for parents and 55.6 (SD 6.0) years old for nonparents (data not shown). The majority of the caregivers were unemployed (52.9%), had received senior middle school education or below (68.9%), and had a monthly household income of over 5000 CNY (60.5%). The mean age of children was 22.4 months old. Approximately half of the children were 25-to-36 months old (53.2%) and boys (52.2%). A total of 81.4% of children were introduced to complementary foods at 6–8 months old. The mean scores of caregivers’ emotional and instrumental feeding, children’s EOE, and EUE were 2.7, 2.1, and 2.9, respectively (Table 1).

### 3.2. Children’s Consumption of UPF

**Figure 2:**
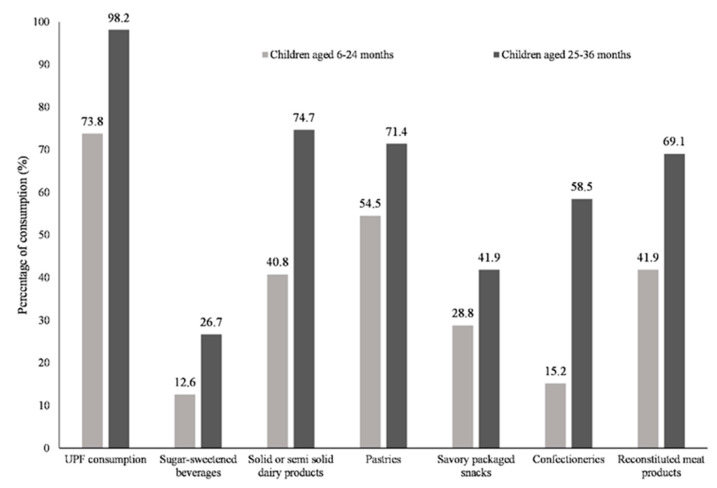
The consumption of UPF by children aged 6–24 months (*n* = 191) and children aged 25–36 months (*n* = 217). UPF: ultra-processed foods. The percentages of consumption of UPF, as well as each UPF item, were different between children aged 6–24 months and children aged 25–36 months (*p* < 0.05).

Among the children, 86.8% consumed UPF. The most frequently consumed UPF were pastries (63.5%), followed by solid or semi-solid dairy products (58.8%), and reconstituted meat products (56.4%) (Table 2). The percentage of UPF consumption was 73.8% for children aged 6–24 months old, and 98.2% for children aged 25–36 months old. With an increase in children’s age, an increasing trend of total UPF consumption and each UPF item were observed (*p* < 0.05) (Figure 2). The median times of the consumption of total UPF, solid or semi-solid dairy products, pastries, and reconstituted meat products per week were 9, 1, 2, and 1, respectively. The median amounts consumed per week were 365 g, 100 g, 30 g, and 19 g, respectively (Table 2).

### 3.3. Associations between Caregivers’ Emotional and Instrumental Feeding and Children’s Consumption of UPF

The univariate associations between participants’ sociodemographic characteristics and children’s consumption of UPF, and the frequencies and the amount of UPF consumption in a week are presented in Tables S2–S4. After controlling for confounders, a higher score of caregivers’ emotional and instrumental feeding was associated with higher odds of children’s consumption of UPF (OR = 1.59, 95% CI: 1.01, 2.49), sugar-sweetened beverages, solid or semi-solid dairy products, pastries, and savoury packaged snacks. Higher scores of children’s EUE were associated with children’s consumption of UPF (OR = 1.61, 95% CI: 1.07, 2.42) and reconstituted meat products. Children’s EOE and their consumption of UPF or each UPF item was not related (Table 3). Higher scores of caregivers’ emotional and instrumental feeding were associated with higher odds of children’s consumption of UPF ≥ nine times/week (OR = 1.80, 95% CI: 1.35, 2.39) and pastries ≥ two times/week. Higher scores of children’s EUE were associated with children’s consumption of UPF ≥ nine times/week (OR = 1.33, 95% CI: 1.03, 1.73) (Table 4). Higher scores of caregivers’ emotional and instrumental feeding were associated with higher odds of children’s consumption of UPF ≥ 365 g/week (OR = 1.85, 95%CI: 1.38, 2.49), solid or semi-solid dairy products ≥ 100 g/week, pastries ≥ 30 g/week, and reconstituted meat products ≥ 19 g/week. Higher scores of children’s EUE were associated with children’s consumption of reconstituted meat products ≥ 19 g/week (Table 5). Children’s EOE and the frequency or amount of UPF consumption in a week was not related (Table 4 and Table 5).

### 3.4. The Effect of Children’s Emotional Eating on the Relationship between Caregiver’s Emotional and Instrumental Feeding and Children’s Consumption of UPF Item

Caregivers’ emotional and instrumental feeding was positively associated with children’s EOE (r = 0.333, *p* < 0.001) and EUE (r = 0.354, *p* < 0.001) (data not shown). Children’s EUE significantly mediated the associations between caregivers’ emotional and instrumental feeding and children’s consumption of reconstituted meat products (effect: 0.026, 95%CI: 0.005, 0.050) (Table 6).

## 4. Discussion

This study reported a high percentage of UPF consumption (86.8%) among children aged 6-to-36 months old in China. The median times and amount of UPF consumption per week were nine times and 365 g, respectively. To our knowledge, this was the first study investigating the associations between caregivers’ emotional and instrumental feeding, children’s emotional eating, and the consumption of UPF among children aged 6-to-36 months old. Caregivers’ emotional and instrumental feeding was positively associated with children’s consumption behaviours of the total UPF, solid and semi-solid dairy products, and pastries. Children’s EUE was positively associated with the consumption and weekly consumption frequency of total UPF, as well as the consumption behaviours of reconstituted meat products. Moreover, children’s EUE mediated the associations between caregivers’ emotional and instrumental feeding and children’s consumption of reconstituted meat products.

The percentage of UPF consumption in our study was high and similar to the results from Brazilian and American cross-sectional studies among infants and toddlers [11,13,34]. The percentages of savoury packaged snack consumption and sugar-sweetened beverage consumption in our study were lower than those in Indonesia (81.6% and 40.0%) [12]. The median times of pastry and reconstituted meat product consumption among our sample were similar to those among children aged two-to-five years old in Hong Kong, China [35]. The median times of pastry consumption among our sample were less than those among children aged one-to-three years old in the Netherlands [33]. It appears that there were geographical and cultural variations in UPF consumption. The mean scores of emotional and instrumental feeding among our study participants were similar to those of parents in three cities (Harbin City, Xiamen City, and Xi’an City, 2.8 ± 0.7) in mainland China [28], and parents in Hong Kong [35].

Similar to studies among older children in the Netherlands, the UK, and the US [17,18,19,20,21], and studies among younger children in the Netherlands and Hong Kong, China [33,35], our study demonstrated that caregivers’ emotional and instrumental feeding was positively associated with children’s UPF consumption and the frequency and amount of weekly consumption. In the context of emotional and instrumental feeding practices, caregivers preferred to offer UPF, such as confectioneries, pastries, and savoury packaged snacks, to comfort children’s distress, and to reward or punish children’s behaviours among children aged 4-to-12 years old in the Netherlands [16]. More studies investigating food types provided in the context of emotional and instrumental feeding in younger children under three years old are needed. Emotional and instrumental feeding might not change children’s preference for rewarded/punished behaviours [36]. However, children might be misled to consume foods owing to their performance and their bad emotions, rather than their own hunger/satiety cues [37,38]. Frequent use of emotional and instrumental feeding might increase children’s intake of UPF. A cross-sectional study in the US showed that children aged three-to-seven years old preferred high fat and sugar foods if parents used emotional or instrumental feeding [39]. Another study among children 6–12 years old demonstrated that caregivers using food as a reinforcer to control children’s behaviour was associated with an increased consumption of that reinforcing food [40]. Caregivers should be advised to avoid using emotional and instrumental feeding strategies.

In our study, children with higher scores of EUE were positively related to UPF consumption and the frequency of UPF consumption. Likewise, children with higher scores of EUE were positively related to reconstituted meat product consumption and their amount of consumption weekly. EUE was children’s coping strategy for negative emotions. When experiencing negative emotions, children were likely to eat less and consume specific UPF, such as reconstituted meat products, to meet their satiety needs. However, a study in the UK showed that in negative mood conditions, children characterised as experiencing EUE consumed less energy from crisps or potato chips [41]. The consumption of chips or crisps appeared to be more associated with good mood and leisurable conditions, rather than negative moods. Our results, together with the above the UK findings, might imply that EUE only affects the intake of specific foods (such as reconstituted meat products), but not the total dietary intake. In the future, the dietary energy or the food type consumed by children in the context of emotional undereating should be investigated to examine this hypothesis.

Our study was consistent with two studies in China showing that caregivers’ emotional and instrumental feeding were positively associated with children’s EUE [42,43]. The reason might be that those feeding practices increase children’s expression of bad moods by avoiding foods [43]. In the context of emotional and instrumental feeding, food serves as a response to negative emotions, as opposed to physiological needs. In the long run, caregivers’ emotional and instrumental feeding practices might impair children’s capability of emotion regulation, and increase children’s emotional eating behaviours [44]. Furthermore, our study was novel in demonstrating a mediation effect of children’s EUE on the association between caregivers’ emotional and instrumental feeding and children’s consumption of reconstituted meat products. It is likely that when exposed to emotional and instrumental feeding, children were feeling negative emotions and tended to eat less. Therefore, they chose reconstituted meat products that have a fast satiety effect to eat less. EUE was more likely to be associated with satisfying satiety rather than enjoying the meal occasion. Further studies to examine the above mechanism are warranted.

Finally, we failed to find a significant association between EOE and UPF consumption, which is similar to a study in the UK that found EOE among children aged 34–59 months old was not related to their snack food intake, regardless of their mood conditions [41]. However, our results contradicted findings among older children aged 9-to-11 years old in other countries [25]. This might be because children’s EOE increased with age. Between the ages of four and ten years old, children’s EOE increased, whereas EUE decreased [23]. The low prevalence of EOE among younger children might contribute to its insignificant association with UPF consumption.

Our findings added to the literature that caregivers’ nonresponsive feeding practices and children’s emotional eating increased children’s UPF consumption. There are some limitations. First, the cross-sectional design made it difficult to draw a causal conclusion. Second, the self-reported feeding and eating behaviours may elicit recall bias. When investigated, caregivers found it difficult to fill in the questionnaire independently due to looking after their children (e.g., soothing the child after vaccination, holding the child, and paying attention to the child’s safety), and the decreased vision or lower education level of elderly caregivers. Therefore, the method of face-to-face interview by trained research staff was used. Although trained research staff reminded the caregiver to feel free and respond as honestly as possible before the interview, the face-to-face interview may result in social desirability bias and reporting bias. Third, our findings might not be generalizable to children in other areas owing to the convenience sampling method.

## 5. Conclusions

The percentage of UPF consumption was high among children under three years of age in the current sample. Caregivers frequently adopting emotional and instrumental feeding practices were positively associated with children’s consumption of UPF, as well as most UPF subgroups. Children’s EUE was positively related to their consumption of reconstituted meat product, as well as a higher weekly amount of reconstituted meat product consumption. Children’s EUE mediated the associations between caregivers’ emotional and instrumental feeding and children’s consumption of reconstituted meat products. Longitudinal studies are warranted to confirm the findings. In the prevention of noncommunicable diseases in childhood and adulthood, caregivers should be educated to avoid emotional and instrumental feeding, and to help children develop healthy eating behaviours and dietary patterns.

## Figures and Tables

**Table 1:** Sociodemographic characteristics of the participants, caregivers’ feeding practices, and children’s emotional eating behaviours (*n* = 408).

Characteristics	*n* (%)/Mean (SD)
Caregiver’s relationship with the child	
Parent	309 (75.7)
Nonparent	99 (24.3)
Caregiver’s education level	
Senior middle school or below	281 (68.9)
College or above	127 (31.1)
Caregiver’s employment status	
Unemployed	216 (52.9)
Employed	192 (47.1)
Caregiver’s weight status †	
Underweight	18 (4.5)
Normal	206 (51.7)
Overweight	116 (29.0)
Obesity	59 (14.8)
Monthly household income	
≤5000 CNY	161 (39.5)
>5000 CNY	247 (60.5)
Child’s age	
6–24 months	191 (46.8)
25–36 months	217 (53.2)
Child’s gender	
Boy	213 (52.2)
Girl	195 (47.8)
Timing of complementary feeding †	
<6 months	62 (15.2)
6–8 months	332 (81.4)
>8 months	13 (3.2)
Caregivers’ emotional and instrumental feeding ‡	2.7 (0.8)
Children’s emotional overeating ‡	2.1 (0.6)
Children’s emotional undereating ‡	2.9 (0.8)

† Variable with missing values. ‡ Assessed by a 5-point Likert scale, ranged from 1 to 5. A higher score indicated a higher frequency of the practice.

**Table 2:** Children’s consumption of UPF (*n* = 408).

UPF Items	Percentage of Consumption *n* (%)	Times of Consumption in a Week		Amount of Consumption in a Week (in g)	
		Median (IQR)	≥Median *n* (%)	Median (IQR)	≥Median *n* (%)
UPF	354 (86.8)	9 (4–14)	210 (51.5)	365.0 (62.0–813.8)	204 (50.0)
Sugar-sweetened beverages	82 (20.1)	0 (0–0)	-	0.0 (0.0–0.0)	-
Solid or semi-solid dairy products	240 (58.8)	1 (0–5)	-	100.0 (0.0–500.0)	220 (53.9)
Pastries	259 (63.5)	2 (0–5)	217 (53.2)	30.0 (0.0–136.3)	205 (50.2)
Savoury packaged snacks	146 (35.8)	0 (0–2)	-	0.0 (0.0–40.0)	-
Confectioneries	156 (38.2)	0 (0–2)	-	0.0 (0.0–20.0)	-
Reconstituted meat products	230 (56.4)	1 (0–3)	-	19.0 (0.0–70.0)	204 (50.0)

UPF: ultra-processed foods; IQR: interquartile range. The estimated density of sugar-sweetened beverages is 1 g/mL. -: Not categorised.

**Table 3:** The associations between children’s UPF consumption and caregivers’ emotional and instrumental feeding, as well as children’s emotional eating (*n* = 408).

Variables	UPF ^a^	Sugar-Sweetened Beverages ^b^	Solid or Semi-Solid Dairy Products ^c^	Pastries ^d^
Emotional and instrumental feeding				
Crude OR (95%CI)	1.37 (0.95,1.98)	1.68 (1.20,2.36) **	1.54 (1.18,2.00) **	1.37 (1.06,1.79) *
Adjusted OR (95%CI)	1.59 (1.01,2.49) *	1.64 (1.17,2.31) **	1.61 (1.21,2.16) **	1.49 (1.12,1.99) **
Emotional overeating				
Crude OR (95%CI)	0.90 (0.58,1.40)	1.05 (0.72,1.53)	0.88 (0.65,1.19)	1.08 (0.79,1.48)
Adjusted OR (95%CI)	1.25 (0.76,2.04)	1.10 (0.74,1.63)	1.04 (0.75,1.46)	1.29 (0.92,1.81)
Emotional undereating				
Crude OR (95%CI)	1.45 (1.03,2.04) *	1.26 (0.92,1.70)	1.21 (0.95,1.54)	1.19 (0.93,1.52)
Adjusted OR (95%CI)	1.61 (1.07,2.42) *	1.24 (0.91,1.69)	1.25 (0.97,1.63)	1.20 (0.93,1.55)
	**Savoury Packaged Snacks ^e^**	**Confectioneries ^f^**	**Reconstituted Meat Products ^g^**	
Emotional and instrumental feeding				
Crude OR (95%CI)	1.60 (1.21,2.11) **	1.27 (0.98,1.66)	1.22 (0.95,1.58)	
Adjusted OR (95%CI)	1.58 (1.20,2.09) **	1.28 (0.96,1.70)	1.31 (0.99,1.74)	
Emotional overeating				
Crude OR (95%CI)	1.12 (0.82,1.53)	0.89 (0.65,1.21)	1.04 (0.77,1.40)	
Adjusted OR (95%CI)	1.20 (0.87,1.66)	1.12 (0.79,1.59)	1.28 (0.91,1.79)	
Emotional undereating				
Crude OR (95%CI)	1.12 (0.87,1.43)	1.14 (0.89,1.46)	1.40 (1.10,1.79) **	
Adjusted OR (95%CI)	1.12 (0.87,1.43)	1.15 (0.88,1.51)	1.46 (1.12,1.90) **	

UPF: ultra-processed foods; OR: odds ratio; CI: confidence interval. ^a^ In the adjusted model (presented by adjusted OR), child’s age, caregiver’s relationship with the child, caregiver’s employment status, and monthly household income were controlled as potential confounders. ^b^ In the adjusted model (presented by adjusted OR), child’s age, caregiver’s weight status, and monthly household income were controlled as potential confounders. ^c^ In the adjusted model (presented by adjusted OR), child’s age, timing of complementary feeding, caregiver’s relationship with the child, and monthly household income were controlled as potential confounders. ^d^ In the adjusted model (presented by adjusted OR), child’s age, child’s gender, timing of complementary feeding, caregiver’s relationship with the child, and monthly household income were controlled as potential confounders. ^e^ In the adjusted model (presented by adjusted OR), child’s age was controlled as the potential confounder. ^f^ In the adjusted model (presented by adjusted OR), child’s age and caregiver’s weight status were controlled as potential confounders. ^g^ In the adjusted model (presented by adjusted OR), child’s age, caregiver’s relationship with the child, caregiver’s education level, employment status, and weight status were controlled as potential confounders. * *p* < 0.05, ** *p* < 0.01.

**Table 4:** The associations between children’s frequencies of UPF consumption and caregivers’ emotional and instrumental feeding, as well as children’s emotional eating (*n* = 408).

Variables	UPF (≥9 times/week) ^a^	Pastries (≥2 times/week) ^b^
Emotional and instrumental feeding		
Crude OR (95%CI)	1.71 (1.31,2.23) ***	1.29 (1.00,1.67) *
Adjusted OR (95%CI)	1.80 (1.35,2.39) ***	1.36 (1.03,1.78) *
Emotional overeating		
Crude OR (95%CI)	1.05 (0.78,1.41)	1.04 (0.77,1.40)
Adjusted OR (95%CI)	1.30 (0.93,1.81)	1.20 (0.87,1.65)
Emotional undereating		
Crude OR (95%CI)	1.29 (1.02,1.64) *	1.03 (0.81,1.31)
Adjusted OR (95%CI)	1.33 (1.03,1.73) *	1.04 (0.81,1.33)

UPF: ultra-processed foods; OR: odds ratio; CI: confidence interval. ^a^ In the adjusted model (presented by adjusted OR), child’s age, caregiver employment status, weight status, and monthly household income were controlled for as potential confounders. ^b^ In the adjusted model (presented by adjusted OR), child’s age, gender, caregiver relationship with the child, weight status, and household monthly income were controlled for as potential confounders. * *p* < 0.05, *** *p* < 0.001.

**Table 5:** The associations between children’s amount of UPF consumption and caregivers’ emotional and instrumental feeding, as well as children’s emotional eating (*n* = 408).

Variables	UPF (≥365 g/week) ^a^	Solid or Semi-Solid Dairy Products (≥100 g/week) ^b^	Pastries (≥30 g/week) ^c^	Reconstituted Meat Products (≥19 g/week) ^d^
Emotional and instrumental feeding				
Crude OR (95%CI)	1.70 (1.30,2.21) ***	1.50 (1.16,1.95) **	1.47 (1.14,1.91) **	1.30 (1.01,1.68)
Adjusted OR (95%CI)	1.85 (1.38,2.49) ***	1.55 (1.16,2.07) **	1.54 (1.16,2.02) **	1.38 (1.04,1.81) *
Emotional overeating				
Crude OR (95%CI)	0.77 (0.57,1.05)	0.89 (0.66,1.20)	0.92 (0.68,1.25)	0.98 (0.72,1.32)
Adjusted OR (95%CI)	0.90 (0.64,1.26)	1.06 (0.76,1.48)	1.11 (0.80,1.53)	1.17 (0.84,1.63)
Emotional undereating				
Crude OR (95%CI)	1.20 (0.94,1.52)	1.19 (0.94,1.51)	1.08 (0.85,1.37)	1.35 (1.06,1.72) *
Adjusted OR (95%CI)	1.25 (0.96,1.63)	1.23 (0.95,1.59)	1.07 (0.84,1.38)	1.37 (1.06,1.78) *

UPF: ultra-processed foods; OR: odds ratio; CI: confidence interval. ^a^ In the adjusted model (presented by adjusted OR), child’s age, caregiver’s relationship with the child, education level, and employment status were controlled as potential confounders. ^b^ In the adjusted model (presented by adjusted OR), child’s age, caregiver’s relationship with the child, and monthly household income were controlled as potential confounders. ^c^ In the adjusted model (presented by adjusted OR), child’s age, caregiver’s relationship with the child, and weight status were controlled as potential confounders. ^d^ In the adjusted model (presented by adjusted OR), child’s age, caregiver’s relationship with the child, education level, employment status, and weight status were controlled as potential confounders. * *p* < 0.05, ** *p* < 0.01, *** *p* < 0.001.

**Table 6:** The mediation effect of children’s emotional undereating on the associations between caregivers’ emotional and instrumental feeding and children’s consumption of reconstituted meat products (*n* = 408).

Path	Indirect Effect Estimate	BootstrappingBias Corrected 95%CI
Emotional and instrumental feeding → Emotional undereating → Reconstituted meat products ^a^	0.026 *	0.005, 0.050
Emotional and instrumental feeding → Emotional undereating → Reconstituted meat products (≥19 g/week) ^b^	0.020	−0.002, 0.040

The calculation of indirect effect estimate used the mediation package in R, and is based on the mediator model and outcome model. In the mediator model, a linear regression model was conducted with emotional undereating as outcome variable, and emotional and instrumental feeding as independent variable. In the outcome model, a logistic regression model was conducted with the consumption of reconstituted meat products as outcome variable, and emotional and instrumental feeding, and emotional undereating as independent variables. CI: confidence interval. ^a^ child’s age, caregiver’s relationship with the child, caregiver’s education level, employment status, and weight status were adjusted in both the mediator and outcome models. ^b^ child’s age, caregiver’s relationship with the child, caregiver’s education level, employment status, and weight status were adjusted in both the mediator and outcome models. * *p* < 0.05.

## Data Availability

The data presented in this study are available on request from the corresponding author.

## Supplementary Materials

The following supporting information can be downloaded at https://www.mdpi.com/article/10.3390/ijerph19084439/s1: Table S1: Scores of scale of items of emotional and instrumental feeding and children’s emotional eating (*n* = 408); Table S2: UPF consumption across different sociodemographic characteristics, by chi-squared test (*n* = 408); Table S3: Frequency of UPF consumption per week across different sociodemographic characteristics, by chi-squared test (*n* = 408); Table S4: Amount of UPF consumption in a week across different sociodemographic characteristics, by chi-squared test (*n* = 408).
