# Supplementary material for: The Associations between Caregivers’ Emotional and Instrumental Feeding, Children’s Emotional Eating, and Children’s Consumption of Ultra-Processed Foods in China
Source: Int J Environ Res Public Health. 2022 Apr 7;19(8):4439. doi: 10.3390/ijerph19084439 (PMC9028148; doi:10.3390/ijerph19084439)
Supplement: Supplementary file 1 [file ijerph-19-04439-s001.zip › ijerph-1662906-supplementary.pdf]

Supplementary Tables

Table S1. Scores of scale of items of emotional and instrumental feeding and children's emotional eating (n = 408).

Items	Mean	SD
**Emotional and instrumental feeding (Cronbach's α: 0.768)**		
When the child becomes restless, irritable or crying, you will give him some foods to make him feel better.	2.9	1.0
When the child is bored or depressed, you will give him some foods, even if you feel he is not hungry.	2.3	1.0
When the child is angry, you will give him some foods to make him feel better.	2.7	1.0
In order to make the child behave, you will promise to give him some foods he likes.	2.8	1.1
When the child behaves well, you will reward him with some foods.	2.9	1.2
**Emotional overeating (Cronbach's α: 0.698)**		
My child eats more when worried.	2.2	0.9
My child eats more when annoyed.	2.1	0.9
My child eats more when anxious.	2.0	0.8
My child eats more when s/he has nothing else to do.	2.3	1.0
**Emotional undereating (Cronbach's α: 0.733)**		
My child eats less when angry.	2.8	1.1
My child eats less when s/he is tired.	2.8	1.1
My child eats more when s/he is happy.	3.2	1.1
My child eats less when upset.	2.6	1.1

Table S2. UPF consumption across different sociodemographic characteristics, by chi-square test (n = 408).

Characteristics	*n*	UPF		Sugar-Sweetened Beverages		Solid or Semi Solid Dairy Products		Pastries		Savoury Packaged Snacks		Confectioneries		Reconstituted Meat Products	
		n (%)	*p*	*n* (%)	*p*	*n* (%)	*p*	*n* (%)	*p*	*n* (%)	*p*	*n* (%)	*p*	*n* (%)	*p*
Caregiver's relationship with the child															
Parent	309	257(83.2)	<0.001	66(21.4)	0.261	175(56.6)	0.112	188(60.8)	0.050	107(34.6)	0.389	114(36.9)	0.324	159(51.5)	<0.001
Nonparent	99	97(98.0)		16(16.2)		65(65.7)		71(71.7)		39(39.4)		42(42.4)		71(71.7)	
Caregiver's education level															
Senior middle school or below	281	247(87.9)	0.314	57(20.3)	0.889	169(60.1)	0.421	180(64.1)	0.719	100(35.6)	0.902	113(40.2)	0.221	169(60.1)	0.022
College or above	127	107(84.3)		25(19.7)		71(55.9)		79(62.2)		46(36.2)		43(33.9)		61(48.0)	
Caregiver's employment status															
Unemployed	216	183(84.7)	0.197	48(22.2)	0.256	122(56.5)	0.308	136(63.0)	0.818	76(35.2)	0.789	77(35.6)	0.254	115(53.2)	0.176
Employed	192	171(89.1)		34(17.7)		118(61.5)		123(64.1)		70(36.5)		79(41.1)		115(59.9)	
Caregiver's weight status †															
Underweight	18	15(83.3)	0.333	0(0.0)	0.035	8(44.4)	0.415	10(55.6)	0.329	6(33.3)	0.288	6(33.3)	0.025	9(50.0)	0.039
Normal weight	206	174(84.5)		47(22.8)		129(62.6)		124(60.2)		68(33.0)		72(35.0)		104(50.5)	
Overweight	116	106(91.4)		27(23.3)		66(56.9)		79(68.1)		50(43.1)		58(50.0)		73(62.9)	
Obesity	59	52(88.1)		7(11.9)		35(59.3)		41(69.5)		19(32.2)		18(30.5)		40(67.8)	
Monthly household income															
≤5000 CNY	161	133(82.6)	0.045	39(24.2)	0.093	84(52.2)	0.028	95(59.0)	0.130	53(32.9)	0.330	58(36.0)	0.458	88(54.7)	0.573
>5000 CNY	247	221(89.5)		43(17.4)		156(63.2)		164(66.4)		93(37.7)		98(39.7)		142(57.5)	
Child' gender															
Boy	213	188(88.3)	0.351	42(19.7)	0.841	131(61.5)	0.251	143(67.1)	0.109	78(36.6)	0.713	78(36.6)	0.483	123(57.7)	0.559
Girl	195	166(85.1)		40(20.5)		109(55.9)		116(59.5)		68(34.9)		78(40.0)		107(54.9)	
Timing of complementary feeding †															
<6 months	62	53(85.5)	0.450	9(14.5)	0.324	35(56.5)	0.151	40(64.5)	0.176	22(35.5)	0.414	22(35.5)	0.217	38(61.3)	0.461
6–8 months	332	288(86.7)		69(20.8)		194(58.4)		214(64.5)		117(35.2)		126(38.0)		183(55.1)	
>8 months	13	13(100.0)		4(30.8)		11(84.6)		5(38.5)		7(53.8)		8(61.5)		9(69.2)	

UPF: ultra-processed foods.
† Variable with missing values.

Table S3. Frequency of UPF consumption per week across different sociodemographic characteristics, by chi-square test (n = 408).

Characteristics	*n*	UPF(≥9 times/week)		Pastries(≥2 times/week)	
		***n* (%)**	***p***	***n* (%)**	***p***
Caregiver’s relationship with the child					
Parent	309	154(49.8)	0.244	158(51.1)	0.142
Nonparent	99	56(56.6)		59(59.6)	
Caregiver’s education level					
Senior middle school or below	281	146(52.0)	0.770	154(54.8)	0.330
College or above	127	64(50.4)		63(49.6)	
Caregiver’s employment status					
Unemployed	216	104(48.1)	0.154	110(50.9)	0.332
Employed	192	106(55.2)		107(55.7)	
Caregiver’s weight status †					
Underweight	18	8(44.4)	0.052	9(50.0)	0.114
Normal weight	206	94(45.6)		98(47.6)	
Overweight	116	70(60.3)		70(60.3)	
Obesity	59	34(57.6)		35(59.3)	
Monthly household income					
≤5000 CNY	161	73(45.3)	0.046	72(44.7)	0.006
>5000 CNY	247	137(55.5)		145(58.7)	
Child’s age					
6 – 24 months	191	64(33.5)	<0.001	88(46.1)	0.007
25–36 months	217	146(67.3)		129(59.4)	
Child’s gender					
Boy	213	113(53.1)	0.504	120(56.3)	0.182
Girl	195	97(49.7)		97(49.7)	
Timing of complementary feeding †					
<6 months	62	36(58.1)	0.404	34(54.8)	0.571
6–8 months	332	166(50.0)		178(53.6)	
>8 months	13	8(61.5)		5(38.5)	

UPF: ultra-processed foods.
† Variable with missing values.

Table S4. Amount of UPF consumption in a week across different sociodemographic characteristics, by chi-square test (n = 408).

Characteristics	n	UPF, ≥365 g/week		Solid or Semi Solid Dairy Products, ≥100 g/week		Pastries, ≥30 g/week		Reconstituted Meat Products, ≥19 g/week	
		n (%)	p	n (%)	p	n (%)	p	n (%)	p
Caregiver's relationship with the child									
Parent	309	144(46.6)	0.015	160(51.8)	0.125	144(46.6)	0.009	141(45.6)	0.002
Nonparent	99	60(60.6)		60(60.6)		61(61.6)		64(64.6)	
Caregiver's education level									
Senior middle school or below	281	151(53.7)	0.025	155(55.2)	0.455	147(52.3)	0.214	148(52.7)	0.109
College or above	127	53(41.7)		65(51.2)		58(45.7)		56(44.1)	
Caregiver's employment status									
Unemployed	216	97(44.9)	0.029	111(51.4)	0.276	103(47.7)	0.273	99(45.8)	0.074
Employed	192	107(55.7)		109(56.8)		102(53.1)		105(54.7)	
Caregiver's weight status†									
Underweight	18	7(38.9)	0.727	7(38.9)	0.353	6(33.3)	0.127	8(44.4)	0.117
Normal weight	206	103(50.0)		118(57.3)		97(47.1)		93(45.1)	
Overweight	116	58(50.0)		59(50.9)		65(56.0)		63(54.3)	
Obesity	59	32(54.2)		34(57.6)		34(57.6)		36(61.0)	
Monthly household income									
≤5000 CNY	161	82(50.9)	0.761	78(48.4)	0.073	75(46.6)	0.232	76(47.2)	0.362
>5000 CNY	247	122(49.4)		142(57.5)		130(52.6)		128(51.8)	
Child's age									
6 – 24 months	191	54(28.3)	<0.001	64(33.5)	<0.001	70(36.6)	<0.001	65(34.0)	<0.001
25–36 months	217	150(69.1)		156(71.9)		135(62.2)		139(64.1)	
Child's gender									
Boy	213	112(52.6)	0.276	121(56.8)	0.222	105(49.3)	0.689	107(50.2)	0.921
Girl	195	92(47.2)		99(50.8)		100(51.3)		97(49.7)	
Timing of complementary feeding†									
<6 months	62	30(48.4)	0.731	35(56.5)	0.217	31(50.0)	0.696	33(53.2)	0.584
6–8 months	332	166(50.0)		175(52.7)		16950.9)		163(49.1)	
>8 months	13	8(81.5)		10(76.9)		5(38.5)		8(61.5)	

UPF: ultra-processed foods.
† Variable with missing values.
